# A Genome-Wide Methylation Study on Essential Hypertension in Young African American Males

**DOI:** 10.1371/journal.pone.0053938

**Published:** 2013-01-10

**Authors:** Xiaoling Wang, Bonita Falkner, Haidong Zhu, Huidong Shi, Shaoyong Su, Xiaojing Xu, Ashok Kumar Sharma, Yanbin Dong, Frank Treiber, Bernard Gutin, Gregory Harshfield, Harold Snieder

**Affiliations:** 1 Georgia Prevention Institute, Department of Pediatrics, Georgia Health Sciences University, Augusta, Georgia, United States of America; 2 Department of Medicine, Thomas Jefferson University, Philadelphia, Pennsylvania, United States of America; 3 The Cancer Research Center, School of Medicine, Georgia Health Sciences University, Augusta, Georgia, United States of America; 4 Center for Biotechnology & Genomic Medicine, School of Medicine, Georgia Health Sciences University, Augusta, Georgia, United States of America; 5 Technology Applications Center for Healthful Lifestyles, Colleges Of Nursing and Medicine, Medical University of South Carolina, Charleston, South Carolina, United States of America; 6 Unit of Genetic Epidemiology and Bioinformatics, Department of Epidemiology, University of Groningen, University Medical Center Groningen, Groningen, The Netherlands; Geisel School of Medicine at Dartmouth, United States of America

## Abstract

**Objective:**

There is emerging evidence from animal studies suggesting a key role for methylation in the pathogenesis of essential hypertension. However, to date, very few studies have investigated the role of methylation in the development of human hypertension, and none has taken a genome-wide approach. Based on the recent studies that highlight the involvement of inflammation in the development of hypertension, we hypothesize that changes in DNA methylation of leukocytes are involved in the pathogenesis of hypertension.

**Method & Results:**

We conducted a genome-wide methylation analysis on 8 hypertensive cases and 8 normotensive age-matched controls aged 14–23 years and performed validation of the most significant CpG sites in 2 genes in an independent sample of 36 hypertensive cases and 60 normotensive controls aged 14–30 years. Validation of the CpG sites in the *SULF1* gene was further conducted in a second replication sample of 36 hypertensive cases and 34 controls aged 15.8–40 years. A CpG site in the *SULF1* gene showed higher methylation levels in cases than in healthy controls in the genome-wide step (p = 6.2×10^−5^), which was confirmed in the validation step (p = 0.011) for subjects ≤30 years old but was not significant for subjects of all ages combined (p = 0.095).

**Conclusion:**

The identification of a difference in a blood leukocyte DNA methylation site between hypertensive cases and normotensive controls suggests that changes in DNA methylation may play an important role in the pathogenesis of hypertension. The age dependency of the effect further suggests complexity of epigenetic regulation in this age-related disease.

## Introduction

Essential hypertension (EH) is a major health problem worldwide with approximately one in three adults suffering from the disease. Although twin and family studies highlight a clear inherited component to EH [1], the current purely sequence-based approach only accounts for a fraction of the genetic risk of the disease as evidenced by the recent genome-wide association studies in which the identified genetic variants explain less than 1% of the blood pressure (BP) variation in the population [2]. Several epidemiological and clinical peculiarities of EH such as the incomplete concordance between monozygotic (MZ) twins (ranges from 38% to 52%) [3], [4] and its late onset and progressive nature, are difficult to explain with traditional DNA sequence-based approaches. These observations may indicate the involvement of epigenetic factors in EH development. Epigenetics refers to all meiotically and mitotically heritable changes in gene expression that are not coded in the DNA sequence itself. DNA methylation is an important epigenetic modification and can play a significant regulatory role in both normal and pathological cellular processes. Emerging evidence from animal studies [5], [6], [7], [8] suggests a key role for methylation in the pathogenesis of EH. However, to date, very few studies [9], [10] have investigated the role of methylation in the development of human EH, and none has taken a genome-wide approach. Based on recent studies [11], [12] that highlight the involvement of inflammation in the development of EH, we hypothesized that changes in the DNA methylation of leukocytes are involved in the pathogenesis of EH. The goal of this study was to characterize the DNA methylation profile in peripheral blood leukocytes in EH cases versus normotensive controls using a 3-stage genome-wide approach.

## Methods

### Subjects

A total of 44 EH cases and 68 age (±2 years) matched controls were selected from 4 existing cohorts in Georgia Prevention Institute, Georgia Health Sciences University using the following inclusion criteria: (1) African American (AA) ancestry; (2) male; (3) having leukocyte DNA available; (4) EH cases have age, sex, and height adjusted systolic BP (SBP) ≥95^th^ percentile (if the age of the subject is less than 20 years), or SBP≥140 mmHg, while controls have age, sex and height adjusted SBP≤20^th^ percentile, or have SBP levels<120 mmHg. These 4 cohorts include the BP stress study (n = 603) [13], the Georgia Cardiovascular twin study (n = 1183) [14], the Lifestyle, Adiposity, and Cardiovascular Health in Youth (LACHY) study (n = 740) [15], and the Prevention of Hypertension in African American Teens (PHAT) study (n = 262) [16]. Both the BP Stress study and the twin study are on-going longitudinal studies which have followed the subjects for more than 10 years. Both studies included roughly equal numbers of AAs and European Americans (EA) or males and females. The BP stress study was established in 1989 with subjects aged 7–16 years at baseline [13] and the Georgia Cardiovascular Twin study was established in 1996 with subjects aged 7–25 years at baseline [14]. LACHY and PHAT are cross-sectional studies. The LACHY study [15]consisted of roughly equal numbers of AA and EA adolescents aged 14–18 years of both sexes and the PHAT study [16] consisted of AA males and females aged 14–20 years. For the subjects from the BP Stress and the Georgia Cardiovascular Twin study, if multiple visits (with multiple leukocyte DNA) were available for a subject, the leukocyte DNA collected at the visit when the subject had the highest (for cases) or lowest (for controls) SBP was used. For the subjects from the twin study, only one twin from a pair was selected if both twin and co-twin met the criteria.

Subjects in all the 4 studies were recruited from Augusta, GA area. For all four cohorts self identification by self-reports of each subject or by a parent if the subject was under 18 years of age was used to classify ethnicity according to previously described criteria [17]. Subjects in all the 4 studies were overtly healthy, free of any acute or chronic illness on the basis of parental reports and were not on anti-hypertensive, lipid lowering, anti-diabetic and anti-inflammatory medications [13], [14], [15], [16]. The Institutional Review Board at the Medical College of Georgia approved the studies. Written informed consent was obtained from all subjects and by parents if subjects were younger than 18 years of age.

The discovery panel included 7 EH cases and 7 age matched controls selected from the 44 EH cases and 68 controls from the Georgia cohorts. To increase the power, we also included 1 AA male MZ pair discordant for EH selected from the Georgia CV twin study in which the EH case had age, sex, and height adjusted SBP≥95^th^ percentile while the control had SBP levels 20 mmHg less than the case.

We included the remaining 37 cases and 61 controls not used in the discovery panel into the first replication panel. However, one EH case and one control failed the pyrosequencing assay. In total, the first replication stage included 36 cases and 60 controls.

The subjects in the second replication panel were selected from two existing cohorts at Thomas Jefferson University, Philadelphia, Pennsylvania. The first cohort included 412 young AA adults aged 18–49 years [18] and the second cohort included 300 AA youth aged 12–19 years [19]. A total of 38 AA male cases (SBP≥140 mmHg or DBP≥90 mmHg with or without medication) and 38 age (±2 years) and BMI (normal/overweight/obese) matched male controls (SBP<120 mmHg and DBP<80 mmHg) were selected from the young adult cohort and 6 AA male EH cases (SBP≥140 mmHg) and 6 age (±2 years) and BMI (normal/overweight/obese) matched controls (SBP<110 mmHg and DBP<70 mmHg) were selected from the youth cohort. Four more controls from the young adult cohort were further included to increase the overall number to 92, which is the number of samples that can be measured by pyrosequencing in one plate. Unfortunately, 22 subjects turned out to have insufficient DNA available to conduct the pyrosequencing. That is, in this 2nd replication cohort data were only available in 70 subjects (63 from the young adult cohort and 7 from the youth cohort).

The participants in both the young adult cohort and the youth cohort were living in urban Philadelphia. All protocols were approved by the Institutional Review Board of Thomas Jefferson University and written informed consent was obtained from each participant at enrollment or from parents if subjects were less than 18 years of age. The participants in the youth cohort were overtly healthy, free of any acute or chronic illness on the basis of parental reports and were not on anti-hypertensive, lipid lowering, anti-diabetic and anti-inflammatory medications. For the young adult cohort, the exclusion criteria included secondary HBP, history of diabetes, renal disease, heart failure, autoimmune disease, sickle cell anemia, or endocrine disorders.

### Measurements

For all the four Georgia cohorts, height and weight were measured by standard methods using a wall-mounted stadiometer and a scale, respectively. Body mass index (BMI) was calculated as weight/height^2^. SBP and diastolic BP (DBP) were measured with Dinamap monitors, using an appropriately sized BP cuff placed on the subject’s right arm. BP measurements were taken at 11, 13, and 15 minutes, during a 15-minute supine relaxation period. The average of the last 2 readings was used to represent SBP and DBP values [13], [14], [15], [16].

Fasting peripheral blood samples in the LACHY cohort and non-fasting peripheral blood samples in the other three cohorts were collected. The buffy coat and plasma samples were separated and stored at −80°C. DNA was extracted from the buffy coat.

For the two cohorts in Pennsylvania, BP was measured, in the seated position, by auscultation. The average of eight separate BP measurements obtained at two separate visits (four measurements at each visit) was used to represent BP values. Fasting peripheral blood samples were collected and DNA was extracted from the buffy coat [18], [19].

### Genome-wide Methylation Chip

The HumanMethylation27 BeadChip from Illumina (Illumina, San Diego, CA, USA) was used. This chip can quantitatively measure 27, 000 CpG sites, covering more than 14,000 well-annotated genes at single-CpG resolution. Each chip can accommodate 12 samples. After bisulfite treatment, 200 ng of the converted DNA was whole genome amplified (WGA) and enzymatically fragmented. The bisulfite-converted WGA-DNA samples were purified and applied to the BeadChips. Image processing and intensity data extraction were performed according to Illumina’s instructions (www.illumina.com/products/infinium_humanmethylation27_beadchip_kits.ilmn). Each methylation data point is represented by fluorescent signals from the methylated and unmethylated alleles. DNA methylation beta values are continuous variables between 0 (completely unmethylated) and 1 (completely methylated), representing the ratio of the intensity of the methylated bead type to the combined locus intensity. Initial array processing and quality control were performed with BeadStudio software. The microarray data discussed in this paper have been deposited in NCBI’s Gene Expression Omnibus and are accessible through GEO Series accession number GSE42774.

### Pyrosequencing

The methylation levels of the top CpG sites from the 2 genes selected for replication were determined by pyrosequencing technology, a rapid and robust method for quantitative methylation analysis. After bisulfite treatment, 10 ng of the converted DNA was used in a PCR reaction to amplify the target region. One of the PCR primers was biotin labeled. Single-stranded biotinylated PCR products were prepared for sequencing by use of the Pyrosequencing Vacuum Prep Tool according to the manufacturer’s instructions. The PCR products (each 10 µl) were sequenced by Pyrosequencing PSQ96 HS System (Pyrosequencing-Qiagen) following the manufacturer’s instructions. The methylation status of each locus was analyzed individually as a T/C SNP using QCpG software (Biotage, Kungsgatan, Sweden). PCR primers and sequencing primers for the 2 genes selected for replication are available upon request.

### Statistical Analysis

To identify genome wide methylation differences between EH cases and controls, the LIMMA (Linear Models for Microarray Analysis) package from the Bioconductor project [20] was used. LIMMA uses an empirical Bayes approach that uses the variability in all genes for testing for significant differences, which results in more stable inferences for a relatively small number of arrays. We used a design matrix of a paired test to analyze each CpG site for differential methylation. Each CpG site was assigned a raw p-value based on a moderated t statistic. To correct for multiple testing, the set of raw p-values were converted to false discovery rates (FDR) according to Benjamini and Hochberg [21]. For the replication cohort, the methylation levels of the CpG sites were square root-transformed to obtain a better approximation of the normal distribution prior to analysis. A Student’s t-test was used to investigate whether their methylation levels differed between cases and controls. Linear regression was further used to adjust for the potential effect of age and BMI. We combined the replication steps as well as the genome wide step on the CpG sites carried to the validation stages with meta-analysis using the weighted z score–based approach implemented in the package METAL [22]. Preliminary analyses, t-tests and regression analyses were done using STATA 8 (StataCorp, College Station, Texas).

Gene ontology analysis was conducted with the FatiGO tool [23]. FatiGO takes two lists of genes and converts them into two lists of GO terms. Then a Fisher's exact test for 2×2 contingency tables is used to check for significant over-representation of GO terms in one of the sets with respect to the other one. Multiple testing correction (indexed by adjusted p values) to account for the multiple hypotheses tested (one for each GO term) was applied to reduce the likelihood of false positives. Since at least two CpG sites were included for the majority of genes in the genome-wide chip, for each gene we only used the CpG site with the lowest p value.

## Results

**Figure 1:**
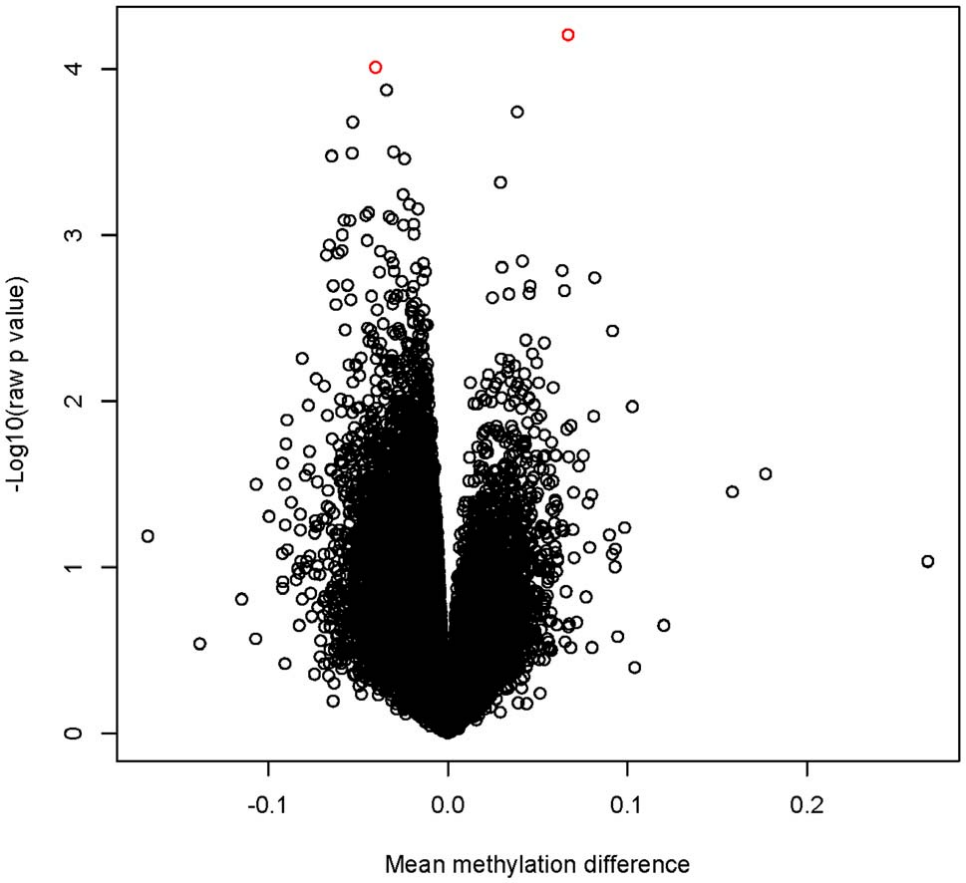
Volcano plot showing raw p values versus mean methylation difference between cases and controls. The two most significant CpG sites are highlighted in red.

Table 1 displays the general characteristics of the 7 EH cases and 7 controls in the discovery panel and Table S1 displays the general characteristics of the 1 MZ pair discordant for EH. Figure 1 is a volcano plot showing the raw p-values for all CpG sites versus mean methylation difference between the case and the control group. We did not observe any CpG sites survive multiple testing corrections with the most significant CpG site showing a FDR of 0.75 and a raw p value of 6.2×10^−5^. Table 2 lists the top 10 most significant CpG sites. Out of the 10 CpG sites, we selected the top 2 CpG sites (one CpG site in the sulfatase 1 gene [*SULF1*] and one CpG site in the prolylcarboxypeptidase gene [*PRCP*]) for validation in the replication cohort. The general characteristics of the first replication cohort are displayed in Table 3. Although the pyrosequencing assays were designed to target one specific CpG site for each gene (Illumina ID cg04845579 for *SULF1* and cg09772827 for *PRCP*), both assays covered several surrounding CpG sites. For the *SULF1* gene, methylation levels on 4 CpG sites were obtained with CpG2 as the target CpG site. All these 4 CpG sites locate in the promoter region with a distance of 214, 186, 119, and 99 base pairs upstream to the transcription starting site. The differences in methylation status of these 4 CpG sites between cases and controls are shown in Table 4. The methylation levels of CpG1 and CpG2 (the target CpG site) were significantly higher in cases than in controls (p = 0.040 and 0.046, respectively). The results remained significant after adjustment for age (p = 0.041 and 0.038, respectively) but became non-significant after further adjustment for BMI (p = 0.074 and 0.081, respectively). For the *PRCP* gene, methylation levels on 8 CpG sites were obtained with CpG6 as the target CpG site. All these 8 CpG sites locate in the first intron with a distance of 293, 302, 305, 320, 323, 346, 353 and 365 base pairs downstream of the transcription starting site. The differences in methylation status of these 8 CpG sites between cases and controls are shown in Table S2. None of these CpG sites showed a significant difference in their methylation levels between cases and controls (p ranged from 0.22–0.87). The correlations within samples among the multiple CpG sites measured within each of these two genes are listed in Tables S3 and S4, respectively.

**Table 1:** General characteristics of cases and controls for genome-wide methylation analysis.

	Cases	Controls
N	7	7
Age, years	18.0±3.1 [14.8–23.3]	18.3±3.2 [14.8–23.0]
BMI, kg/m^2^	27.3±3.5 [21.2–31.5]	23.8±5.5 [17.8–33.2]
SBP, mmHg	145.8±4.7 [137.3–149.5]	107.6±3.3 [103.3–114]
SBP percentile	0.99±0.01 [0.96–1.0]	0.16±0.04 [0.10–0.19]
DBP, mmHg	69.1±6.8 [62.0–78.3]	55.4±5.4 [49.5–63.3]
DBP percentile	0.45±0.22 [0.25–0.89]	0.14±0.10 [0.01–0.26]

Means±SD [Range].

**Table 2:** Top 10 differentially methylated CpG sites.

Gene	Illumina ID	Distance to TSS	CpG island	Methylation, %			P	FDR
				Case	Control	Difference		
SULF1	cg04845579	186	NO	29.49	22.79	6.70	0.000062	0.75
PRCP	cg09772827	346	YES	13.85	17.87	−4.03	0.000098	0.75
NEUROG1	cg14958635	–	YES	7.82	11.24	−3.41	0.000134	0.75
PITPNA	cg11719157	630	YES	61.38	57.51	3.87	0.000182	0.75
SLC26A10	cg14371590	222	NO	22.04	27.33	−5.30	0.000209	0.75
CDC34	cg27431859	691	YES	10.43	13.45	−3.02	0.000315	0.75
C9orf95	cg07962315	1375	NO	37.50	42.83	−5.33	0.000321	0.75
YWHAQ	cg06701500	565	YES	13.79	20.26	−6.47	0.000334	0.75
SIRT7	cg15118204	191	YES	19.48	21.89	−2.41	0.000348	0.75
CLDN5	cg04463638	148	NO	75.20	72.27	2.93	0.000483	0.75

TSS, transcription starting site.

**Table 3:** General characteristics of the subjects in GA cohort (1^st^ replication panel).

	Case	Control
N	36	60
Age, years	20.6±5.3 [14.3–30.7]	19.6±4.5 [14.1–30.9]
BMI, kg/m^2^	29.9±8.7 [21.0–52.4]	24.7±8.0 [16.5–59.9]
SBP, mmHg	143.6±7.5 [133.3–175]	106.9±5.2 [93.7–117]
SBP percentile	0.98±0.01 [0.96–1.00]	0.12±0.06 [0.02–0.20]
DBP, mmHg	71.9±10.4 [56.5–96.5]	58.6±5.6 [46–74.5]
DBP percentile	0.50±0.23 [0.18–0.98]	0.20±0.13 [0.03–0.67]

Means±SD [Range].

**Table 4:** Pyrosequencing results of SULF1 gene in GA cohort.

	Case	Control	P	P, adjusted^a^	P, adjusted^b^
CpG1	16.8±9.0	13.4±7.5	0.040	0.041	0.074
CpG2	22.7±9.4	19.0±8.6	0.046	0.038	0.081
CpG3	5.94±3.04	4.8±3.22	0.105	0.098	0.204
CpG4	20.6±9.1	17.8±7.9	0.106	0.101	0.238

a Adjusted for age.

b Adjusted for age and BMI.

The CpG sites in the SULF1 gene were further validated in the second replication panel. The general characteristics of this cohort are displayed in Table 5. To keep the second comparable to the first replication which only comprised subjects ≤30 years old, we further split the sample by age (≤30 years or >30 years). The general characteristics of the split samples were also listed in Table 5. The differences in methylation status of these 4 CpG sites in the SULF1 gene between cases and controls in the second replication panel are shown in Table 6. None of these 4 CpG sites showed a significant difference in their methylation levels between cases and controls either in the overall sample or in the samples split by age. Meta-analysis on the CpG1 and CpG2 with the two replication panels was conducted and the results are shown in Table 7. Significant higher methylation levels of CpG1 & CpG2 were observed in cases (p = 0.014 and p = 0.011, respectively) in the meta-analysis on the first replication cohort and the young age group of the second replication cohort. The significant result remained after adjustment of age (p = 0.017 and p = 0.015, respectively) or age and BMI (p = 0.030 and p = 0.037, respectively). Further meta-analysis with the discovery panel on CpG2 showed a p value of 0.0051 (with p = 0.0027 after adjustment of age and p = 0.0171 after adjustment of age and BMI) in all the subjects and a p value of 0.0004 (with p = 0.0004 after adjustment of age and p = 0.0054 after adjustment of age and BMI) in subjects younger or equal to 30 years old.

**Table 5:** General characteristics of the subjects in PA cohort (2nd replication panel).

	All subjects		Subjects older than 30		Subjects younger or equal to 30	
	Case	Control	Case	Control	Case	Control
N	36	34	31	25	5	9
Age, years	39.3±7.6	35.6±9.7	41.8±3.8	40.8±3.7	23.6±6.2	21.1±5.1
Age range, years	16.8–49	15.8–47	33–49	34–47	16.8–30	15.8–28
BMI, kg/m^2^	29.5±6.2	28.5±5.7	29.4±6.2	28.4±5.6	30.4±7.0	28.9±2.1
SBP, mmHg	149.5±16.3	108.3±6.4	149.8±17.4	108.1±6.7	147.6±5.6	109.0±2.0
DBP, mmHg	94.6±12.3	68.1±6.1	96.8±10.3	69±6.1	80.9±16.2	65.7±6.0
Antihypertensive Medication	25%	0%	29%	0%	0%	0%

Means±SD.

**Table 6:** Pyrosequencing results of SULF1 gene in the PA cohort.

	Case	Control	P		P, adjusted^a^	P, adjusted^b^
**Overall**						
CpG1	12.5±6.2	12.1±5.6	0.77	0.61		0.65
CpG2	19.7±7.0	18.9±6.7	0.64	0.51		0.53
CpG3	5.3±2.1	5.6±1.9	0.59	0.77		0.69
CpG4	20.9±7.3	21.0±7.0	0.97	0.94		0.96
**Older than30**						
CpG1	11.9±6.4	12.0±5.9	0.81	0.88		0.80
CpG2	19.1±7.3	18.6±7.3	0.85	0.81		0.86
CpG3	5.1±2.1	5.6±2.2	0.39	0.41		0.35
CpG4	20.3±7.3	21.1±7.9	0.70	0.70		0.66
**Younger or equal to 30**						
CpG1	16.3±2.7	12.5±4.9	0.13	0.18		0.16
CpG2	23.6±3.3	19.7±4.6	0.12	0.20		0.25
CpG3	6.9±1.9	5.7±1.0	0.13	0.17		0.22
CpG4	24.9±6.4	20.8±4.2	0.17	0.23		0.21

a Adjusted for age.

b Adjusted for age and BMI.

**Table 7:** Meta -analysis on two CpG sites in the SULF1 gene.

		P	P, adjusted^a^	P, adjusted^b^
CpG1	GA cohort+PA cohort	0.080	0.059	0.098
	GA cohort+PA cohort (age≤30)	0.014	0.017	0.030
CpG2	GA cohort+PA cohort	0.095	0.058	0.105
	GA cohort+PA cohort (age≤30)	0.011	0.015	0.037
	Discovery+Replication	0.0051	0.0027	0.0171
	Discovery+Replication (age≤30)	0.0004	0.0004	0.0054

a Adjusted for age.

b Adjusted for age and BMI.

Gene Ontology analysis was performed to test whether some common functional trends in molecular functions and biological processes were associated with the genes exhibiting differences between cases and controls for the genome-wide methylation analyses. We included those genes with a raw p≤0.01 in the first list (n = 226) and included all the other genes in the second list. As expected from a pilot study in 8 cases and 8 controls, we did not observe any GO categories survive multiple testing. Table S5 list the GO categories with raw P value less than 0.05. Interestingly, we observed enriched functional processes that are potentially relevant for inflammation with response to biotic stimulus (GO:0009607), response to other organism (GO:0051707), interleukin-1 production (GO:0032612), and interleukin-13 production (GO:0032616) among the top GO categories. The results are consistent with the involvement of inflammation and oxidative stress in the development of EH.

## Discussion

In this study we aimed to identify methylation differences in peripheral blood leukocytes between EH cases and controls using a genome-wide approach in male AA youth and young adults. We observed increased methylation levels at two CpG sites in the *SULF1* gene in EH cases in comparison with normotensive controls in subjects equal or younger than 30 years.

Our study is the first genome wide methylation study on EH. In fact, there are very few human studies that explored the role of epigenetics on the risk of EH. In one study, Friso et al [9] measured promoter methylation of the *HSD11B2* gene in peripheral blood mononuclear cells from patients with EH and 32 subjects on prednisone therapy. Elevated *HSD11B2* promoter methylation was associated with decreased HSD11B2 activity and EH development in glucocorticoid-treated patients. In a recent study by Smolarek et al [10] global DNA methylation level indexed by the genome level of 5-methylcytosine was significantly lower in patients with EH in comparison with controls.

The protein encoded by the *SULF1* gene is Sulfatase 1 (Sulf1). It is a cell surface polypeptide that can rapidly modify the sulfation status of heparin sulfate proteoglycans (HSPGs), resulting in changes in HSPG-related signal transduction pathways [24]. Sulf1 has been reported to be down-regulated in several human cancers [25], [26]. Absence of Sulf1 in cancer cell lines is associated with increased cell growth, proliferation and reduced cell apoptosis [25]. In addition to cancer, Sulf1 was also studied with respect to normal development including neural, muscular, vascular and skeletal development. However, there is no direct study on Sulf1 and EH. *SULF1* and *SULF2* (another protein in this family) double knockout mice show impairment in skeletal development [27], but whether they display high blood pressure has never been explored.

In this study, we observed that the methylation levels of 2 CpG sites in the promoter region of the *SULF1* gene were higher in EH cases than in controls. This is in line with the previous studies [28], [29] in cancers in which epigenetic silencing is involved in the down-regulation of Sulf1. The *SULF1* gene spans a ∼211 kb genomic fragment on chromosome 8q13.3 with 23 exons [25]. Staub et al. [29] observed that methylation of 12 CpG sites within *SULF1* exon 1A was associated with ovarian cancer cells and primary ovarian cancer tissues lacking Sulf1 expression. This region is about 10 kb downstream of the promoter region we targeted. The relationship between the methylation level of this region and the promoter region is unknown. No CpG island exists in this gene and the two CpG sites showing significant association with EH in our study locate at a distance of 214bp and 186 bp upstream from the transcription start site. There is a possibility that methylation of these two CpG sites or other CpG sites with methylation levels correlated with them inhibits the interactions between DNA sequence and nuclear proteins, resulting in changes in gene expression. In-silico analysis of the region of these two CpG site using TFSEARCH software [30] did not find they located at any known transcription factor binding sites. However, methylation of these two CpG sites may suppress gene transcription by recruiting methylcytosine-binding proteins that in turn associate with large protein complexes containing corepressors and histone deacetylases. The binding of these complexes to DNA may lead to a change in the chromatin structure from an active to an inactive form [31]. This speculation needs to be confirmed.

The age dependency of the effect of *SULF1* gene methylation on EH may be related to the different pathogenesis of EH in youth in comparison with middle aged or older people. For examples, sympathetic nervous system hyperactivity is most apparent in youth with EH while abnormal development of aortic elasticity or reduced development of the microvascular network is more related to high BP later in life [32]. Furthermore, there is evidence showing that BP regulation may be controlled by different set of genes at different ages [1]. Two longitudinal twin studies [33], [34] have observed age-specific genetic variation for blood pressure, this is, there are new genes being switched on or off at different time points. On the other hand, it is also possible that the current study did not have enough power to find the effect of methylation on EH in middle aged or older people in consideration of the fact that disease-specific epigenetic alterations may be masked by the background of age-related and medication-arising epigenetic “drift” [35], [36].

The observed DNA methylation differences between EH cases and controls were relatively small. They were 6.7% in the genome-wide step and 4.1% in the replication step for the *SULF1* gene CpG2. This modest level of differences raises an important question: what is the biological significance of changes in methylation to this degree? Although transcriptional profiling studies will be very valuable in understanding this question, cellular RNA is not available for the samples used in the current study, which were selected from several existing cohorts. We searched the GEO database and identified a dataset [37] (GSE3846) which included genome wide gene expression data in peripheral blood samples in six healthy volunteers tested by Affymetrix microarrays. In all the 6 samples, the expression of SULF1 was detectable. The average expression value of SULF1 is 4.38 at baseline. The average rank order of expression measurement in each subject is 69%, which means that about 31% genes have lower gene expression levels compared with SULF1 in peripheral blood samples of healthy individuals. This confirms that the SULF1 gene is expressed in peripheral blood cells. However, we failed to find any GEO dataset which included both methylation and gene expression data from peripheral blood samples. Therefore, we cannot test whether DNA methylation in *SULF1* affects its expression in peripheral blood cells.

In this study, we used the DNA from leukocytes, which represent different cell populations with distinct epigenetic profiles [38]. A recent study by Kelsey’s group [39] indicated that shifts in leukocyte subpopulations may account for a considerable proportion of variability in peripheral blood DNA methylation of diseases such as head and neck squamous cell carcinoma and ovarian cancer. This study also provided a list of the top 50 differentially methylated CpG sites among 6 leukocyte subtypes including CD4^+^ T cells, CD8^+^ T cells, CD56^+^ NK cells, B cells, monocytes and granulocytes. The SULF1 gene CpG site is not within the list. Another study [40] from the same group developed an algorithm which predicts the distributions of the 6 leukocyte subtypes using illumina 27 k methylation data from peripheral blood DNA. We applied this algorithm to our data and did not observe difference in the distributions of these 6 cell types between EH cases and controls (Table S6). Therefore, it is highly unlikely that our significant finding on the *SULF1* gene is caused by shifts in these 6 leukocyte subpopulations. On the other hand, it is plausible that only the change in the epigenetic profile of one specific cell type is related to EH. In this case, the actual epigenetic differences might be more substantial than reported here but only present in this specific blood leukocyte cell type. Future studies on epigenetic profiling of various types of cell populations of leukocytes are warranted to gain a greater understanding of the epigenetic dysregulation in EH.

Two strengths of our study deserve mentioning. First, we selected controls with low blood pressure, which maximizes the power to make discoveries. Second, a hypothesis free genome-wide approach was used. This approach supersedes the limitations imposed by candidate gene methylation studies and allows searching the whole genome in an unbiased manner.

Interpretation of these data is also limited by several additional constraints. First, in this study we aimed to identify EH related methylation changes. However, our study design cannot determine whether the identified methylation changes are the cause or the consequence of EH. Future studies on subjects with baseline DNA and follow-up for *de novo* development of EH will be needed to resolve causality [41]. Second, because obesity is an important risk factor for EH, patients often have higher BMI levels than normotensive controls. Obesity might be a confounder explaining the relation between methylation levels of the *SULF1* gene and EH. In the first replication cohort, the association of the *SULF1* gene CpG1 and CpG2 with EH attenuated and became non-significant after adjustment of BMI. Therefore, in the second replication cohort, controls were selected to match with cases on obesity status (normal weight/overweight/obese). We could not replicate the findings from the first 2 stages in the overall analysis. In addition to BMI, this discrepancy might also be due to the age difference between the second replication cohort and the cohorts used in the first 2 stages. Moreover, it is also possible that *SULF1* gene methylation is a mediator of obesity related EH. In this case, including BMI as a covariate in the analysis or matching cases and controls on obesity status will lead to over-adjustment. Future studies on hypertensive subjects with normal weight are needed to clarify whether *SULF1* gene methylation independently affects EH. Third, in the current study, the Infinium HumanMethylation27 Beadchip was selected because of its quantitative measure at each CpG site. However, the limited coverage of this genome-wide chip will restrict the findings to certain CpG sites within certain genes. Future studies should use chips with more complete coverage of the genome such as the recently released 450 K Infinium Methylation BeadChip from Illumina. Fourth, the current study is a pilot study with the genome-wide step conducted only in 7 EH cases and 7 normotensive controls and one MZ pair discordant for EH. Future studies with much larger sample size are warranted to discover a more complete profile of EH related methylation changes.

In conclusion, we identified a reproducible change in DNA methylation of peripheral blood leukocytes between EH cases and controls in subjects ≤30 years. It provides preliminary evidence that DNA methylation may play an important role in the pathogenesis of EH. Further studies are warranted to determine the causal direction of this relationship.

## Supporting Information

Table S1 General characteristics of the MZ pair discordant for EH.
(DOCX)

Table S2 Replication results for PRCP gene.
(DOCX)

Table S3 Correlation among the CpG sites in the *SULF1* gene.
(DOCX)

Table S4 Correlation among the CpG sites in the *PRCP* gene.
(DOCX)

Table S5 Gene-Ontology analysis.
(DOCX)

Table S6 Cell population estimates in cases vs. controls.
(DOCX)
