# Supplementary material for: A Genome-Wide Methylation Study on Essential Hypertension in Young African American Males
Source: PLoS One. 2013 Jan 10;8(1):e53938. doi: 10.1371/journal.pone.0053938 (PMC3542324; doi:10.1371/journal.pone.0053938)
Supplement: Table S1 — General characteristics of the MZ pair discordant for EH. (DOCX) [file pone.0053938.s001.docx]

| Table S1. General Characteristics of the MZ pair discordant for EH | | |
| --- | --- | --- |
|  | Case | Control |
| Age | 18.9 | 18.9 |
| BMI, kg/m^2^ | 24.78 | 26.08 |
| SBP, mmHg | 147 | 124 |
| SBP percentile | 0.997 | 0.735 |
| DBP, mmHg | 62 | 62 |
| DBP percentile | 0.196 | 0.185 |
