# Supplementary material for: A Genome-Wide Methylation Study on Essential Hypertension in Young African American Males
Source: PLoS One. 2013 Jan 10;8(1):e53938. doi: 10.1371/journal.pone.0053938 (PMC3542324; doi:10.1371/journal.pone.0053938)
Supplement: Table S2 — Replication results for PRCP gene. (DOCX) [file pone.0053938.s002.docx]

|  | Control | Case | P |
| --- | --- | --- | --- |
| CpG1 | 7.05 | 7.23 | 0.73 |
| CpG2 | 4.70 | 5.08 | 0.26 |
| CpG3 | 1.40 | 1.21 | 0.57 |
| CpG4 | 1.61 | 1.40 | 0.41 |
| CpG5 | 1.31 | 1.02 | 0.39 |
| CpG6 | 2.62 | 2.32 | 0.22 |
| CpG7 | 1.48 | 1.47 | 0.98 |
| CpG8 | 1.41 | 1.32 | 0.67 |

Table S2. Replication results for PRCP gene
