# Supplementary material for: A Genome-Wide Methylation Study on Essential Hypertension in Young African American Males
Source: PLoS One. 2013 Jan 10;8(1):e53938. doi: 10.1371/journal.pone.0053938 (PMC3542324; doi:10.1371/journal.pone.0053938)
Supplement: Table S3 — Correlation among the CpG sites in the SULF1 gene. (DOCX) [file pone.0053938.s003.docx]

| Table S3: Correlation among the CpG sites in the *SULF1* gene | | | | | |
| --- | --- | --- | --- | --- | --- |
|  | CpG1 | CpG2 | CpG3 | CpG4 |  |
| CpG1 | 1 |  |  |  |  |
| CpG2 | 0.9290 | 1 |  |  |  |
| CpG3 | 0.7476 | 0.7793 | 1 |  |  |
| CpG4 | 0.9487 | 0.9211 | 0.7773 | 1 |  |
| All correlations have p< 0.001 | | | | |  |
