# Supplementary material for: A Genome-Wide Methylation Study on Essential Hypertension in Young African American Males
Source: PLoS One. 2013 Jan 10;8(1):e53938. doi: 10.1371/journal.pone.0053938 (PMC3542324; doi:10.1371/journal.pone.0053938)
Supplement: Table S4 — Correlation among the CpG sites in the PRCP gene. (DOCX) [file pone.0053938.s004.docx]

| Table S4: correlation among the CpG sites in the *PRCP* gene | | | | | | | | |
| --- | --- | --- | --- | --- | --- | --- | --- | --- |
|  | CpG1 | CpG2 | CpG3 | CpG4 | CpG5 | CpG6 | CpG7 | CpG8 |
| CpG1 | 1 |  |  |  |  |  |  |  |
| CpG2 | 0.5673 | 1 |  |  |  |  |  |  |
| CpG3 | 0.2857 | 0.3817 | 1 |  |  |  |  |  |
| CpG4 | 0.5212 | 0.5068 | 0.6613 | 1 |  |  |  |  |
| CpG5 | 0.2651 | 0.3208 | 0.8237 | 0.6769 | 1 |  |  |  |
| CpG6 | 0.3492 | **-0.0129** | 0.2745 | 0.3906 | 0.4100 | 1 |  |  |
| CpG7 | 0.3476 | **0.0346** | 0.3723 | 0.2657 | 0.3908 | 0.5567 | 1 |  |
| CpG8 | 0.4716 | 0.2183 | 0.3642 | 0.4513 | 0.4043 | 0.5638 | 0.3790 | 1 |
| Bolded correlations have p> 0.05 | | | | |  |  |  |  |
