# Supplementary material for: A Genome-Wide Methylation Study on Essential Hypertension in Young African American Males
Source: PLoS One. 2013 Jan 10;8(1):e53938. doi: 10.1371/journal.pone.0053938 (PMC3542324; doi:10.1371/journal.pone.0053938)
Supplement: Table S5 — Gene-Ontology analysis. (DOCX) [file pone.0053938.s005.docx]

| Table S5. Gene-Ontology analysis | | |  |  |
| --- | --- | --- | --- | --- |
|  | GO. ID | Term | P value | FDR |
| Biological process | GO:0065009 | regulation of a molecular function | 0.007 | 0.314 |
|  | GO:0009607 | response to biotic stimulus | 0.009 | 0.314 |
|  | GO:0050790 | regulation of catalytic activity | 0.004 | 0.886 |
|  | GO:0051707 | response to other organism | 0.019 | 1.000 |
|  | GO:0043616 | keratinocyte proliferation | 0.030 | 1.000 |
|  | GO:0032612 | interleukin-1 production | 0.045 | 1.000 |
|  | GO:0032616 | interleukin-13 production | 0.045 | 1.000 |
| Molecular function | GO:0004857 | enzyme inhibitor activity | 0.018 | 1.000 |
|  | GO:0019904 | protein domain specific binding | 0.023 | 1.000 |
|  | GO:0005484 | SNAP receptor activity | 0.031 | 1.000 |
|  | GO:0032052 | bile acid binding | 0.031 | 1.000 |
|  | GO:0030414 | protease inhibitor activity | 0.042 | 1.000 |
|  | GO:0016651 | oxidoreductase activity, acting on NADH or NADPH | 0.044 | 1.000 |
