# Supplementary material for: A Genome-Wide Methylation Study on Essential Hypertension in Young African American Males
Source: PLoS One. 2013 Jan 10;8(1):e53938. doi: 10.1371/journal.pone.0053938 (PMC3542324; doi:10.1371/journal.pone.0053938)
Supplement: Table S6 — Cell population estimates in cases vs. controls. (DOCX) [file pone.0053938.s006.docx]

| Table S6. Cell population estimates in cases vs. controls | | | | | |
| --- | --- | --- | --- | --- | --- |
|  | Est | SE_0_ | SE_1_ | SE_2_ | P-value |
| <Intercept> | -0.15 | 0.77 | 1.42 | 1.43 | 0.918 |
| B cell | -1.36 | 3.64 | 4.14 | 3.58 | 0.704 |
| Granulocyte | -2.52 | 3.63 | 6.72 | 5.75 | 0.662 |
| Monocyte | -0.98 | 1.04 | 1.67 | 1.62 | 0.547 |
| NK | -0.98 | 0.55 | 1.36 | 1.36 | 0.471 |
| T Cell (cd4+) | -3.87 | 0.93 | 2.04 | 1.99 | 0.052 |
| T Cell (cd8+) | 9.46 | 0.92 | 7.67 | 7.61 | 0.214 |
| Est = Regression coefficient estimate (× 100%) | | | | | |
| SE_0_ = Naïve standard error (× 100%) | | | | | |
| SE_1_ = Single-bootstrap standard error (× 100%) | | | | | |
| SE_2_ = Double-bootstrap standard error (× 100%) | | | | | |
| P-values were computed using SE_2_ | | | | | |
